# Ultra-early changes in vascular parameters from dynamic contrast enhanced MRI of breast cancer xenografts following systemic therapy with doxorubicin and liver X receptor agonist

**DOI:** 10.1186/s40644-019-0280-y

**Published:** 2019-12-19

**Authors:** Kathinka E. Pitman, Kine M. Bakke, Alexandr Kristian, Eirik Malinen

**Affiliations:** 1 Department of Physics, University of Oslo, P.O. Box 1048 Blindern, 0316 Oslo, Norway; 2 Department of Oncology, Akershus University Hospital, Lørenskog, Norway; 3 Department of Tumour Biology, Oslo University Hospital, Oslo, Norway; 4 Department of Medical Physics, Oslo University Hospital, Oslo, Norway

**Keywords:** Cancer, Xenograft, Mouse model, Contrast agent, Pharmacokinetic modelling, Doxorubicin, LXR agonist

## Abstract

**Background:**

Dynamic contrast enhanced magnetic resonance imaging (DCE-MRI) may be used to depict tumour vascular structure and for therapy response assessment in various tumour sites. The purpose of the current work is to examine whether ultra-early changes in tumour physiology following cytotoxic treatment with doxorubicin and liver X receptor (LXR) agonist GW3965 are detectable by DCE-MRI.

**Methods:**

36 female, athymic nude foxn1nu mice with bilaterally implanted breast cancer xenografts (17 with ER-positive HBCx34, 19 with triple-negative HBCx39) were randomised in the following treatment groups; control, GW3965 (40 mg/kg p.o.), doxorubicin (8 mg/kg i.v.) and a combination therapy of GW3965 and doxorubicin. DCE-MRI (3D FLASH on a 7 T preclinical scanner) was performed at baseline and one and six days after onset of treatment. Wash-in (30 s p.i.) and wash-out (300 s p.i.) enhancement were quantified from dynamic uptake curves, before voxel-by-voxel fitting to the pharmacokinetic Tofts model and generation of maps for the resulting parameters *K*^trans^, ν_e_ and ν_B_. Treatment effect was evaluated by univariate repeated measures mixed-effects maximum likelihood regression models applied to median tumour data.

**Results:**

We found no effects of any treatment 24 h post treatment. After 6 days, doxorubicin given as both mono- and combination therapy gave significant increases of ~ 30% in wash-in enhancement (*p* < 0.011) and *K*^trans^ (*p* < 0.017), and 40–50% in ν_B_ (*p* < 0.024) for HBCx34, but not for HBCx39. No effects of GW3965 were observed at any time (*p* > 0.1).

**Conclusions:**

Twenty-four h after onset of treatment was too early to evaluate treatment effects by DCE-MRI. Early enhancement and *K*^trans^ were approximately equally sensitive metrics to capture treatment effects six days pt. Pharmacokinetic modelling however allowed us to attribute the observed effect to changes in tumour perfusion rather than increased retention.

## Introduction

Obesity and metabolic syndrome are increasingly important risk factors for breast cancer, in particular in post-menopausal women. Oestrogen receptor (ER) positive breast cancer accounts for roughly 70% of breast cancer cases. Cholesterol metabolism is frequently altered in cancer, and is associated with increased proliferation and angiogenesis [1, 2].

Neoadjuvant chemo- and endocrine therapy is the standard of care for locally advanced breast cancers, aiming to improve patient outcome and maximise breast conserving surgery. A subset of patients however, does not respond to the assigned therapy. Early identification of non-responders may allow swift intervention and reassignment to a different treatment regime, improving survival while saving both time and money spent on ineffective therapy.

Functional imaging may be used for non-invasive serial assessment of treatment response at several time points. Dynamic contrast enhanced magnetic resonance imaging (DCE-MRI) has shown promise in the early prediction of pathological response to neoadjuvant chemotherapy in breast cancer [3]. Here, MR-signal enhancement after injection of a paramagnetic contrast agent (CA) reflects characteristics of tumour vascularity such as perfusion, microvascular permeability, and extracellular extravascular volume fraction [4]. Physiological changes occur upstream of changes in morphology, and therefore allow earlier assessment of treatment response compared with traditional tumour volume measurements [5].

Doxorubicin is the most well-known anthracycline used clinically in the treatment of breast cancer and has well-established cytotoxic effect [6], as well as a more recently pin-pointed anti-angiogenic effect [7]. Given that doxorubicin has fast-occurring effects on tumour vasculature [8], we wished to investigate whether ultra-early changes (24 h and 6 days) after administration of doxorubicin were detectable by DCE-MRI. We have previously seen significant changes in ^18^F-FDG-PET K_1_ 24 h after radiotherapy [9] and antiangiogenic treatment [10] of tumour xenografts. We further wanted to investigate whether we also could detect synergistic effects of a more subtly acting targeted agent, LXR-agonist GW3965. LXR-activation normalises cholesterol homeostasis and has cytostatic and anti-angiogenic effect in breast tumours [11]. LXR-activation moreover has added action on ER^+^ breast cancers by modulating levels of available oestrogen precursors [12]. We therefore selected an ER^+^ and a triple-negative xenograft model to elucidate whether DCE-MRI could be useful in monitoring treatment induced changes very early after onset of therapy.

## Materials and methods

### Xenograft tumour models

36 female, nude athymic Foxn1^nuu^ mice bilaterally implanted with two different breast cancer xenografts were included; one triple-negative (HBCx39, *n* = 19) and one oestrogen receptor positive (ER^+^) invasive ductal carcinoma (HBCx34, *n* = 17). All experimental protocols were approved by the National Animal Research Authority and conducted in accordance with the guidelines of the Federation of European Laboratory Animal Science Association (FELASA).

Tumour fragments (~ 1 mm^3^) from previous passage were implanted in the mammary fat pads of mice (7–8 weeks old, mean weight 24.4 g). Gas anaesthesia (4.0% Sevoflurane in O_2_, Baxter, IL, USA) was used for all procedures, including imaging. Tumour volume and body weight was measured every second day in the weeks coming up to and during treatment and monitoring. Tumour volume was estimated as (π/6)*length*width^2^, where width is the size along the axis orthogonal to the longest axis. Fodder and distilled tap water supplemented with 17-β-estradiol (4 mg/ml) was provided ad libitum.

### Treatment with Dox and LXR agonist GW3965

Treatment and monitoring commenced once tumours reached a diameter of 7–10 mm in the longest axis. Calliper measured median (range) tumour volumes of 284 (97–1276) mm^3^ for HBCx34 and 442 (110–1047) mm^3^ for HBCx39 were obtained at baseline. Mice were randomised in four groups; control (no treatment), GW3965 (40 mg/kg p.o., twice daily), Dox (8 mg/kg i.v., single dose) or a combination treatment of GW3965 and Dox (Dox + GW3965). Treatment lasted until the final day of imaging, when animals were sacrificed by cervical dislocation.

### MRI

MRI was performed on a Bruker Biospec 7.05 T bore magnet (Bruker Biospin, Ettlingen, Germany), equipped with a quadrature volume coil. Scans were performed at baseline, one and six days after onset of treatment.

Mice were anaesthetised and catheterised in the tail vein before being placed with tumours in the isocentre of the bore. Respiration rate was monitored using an abdominal pressure sensitive probe, connected to a monitoring and gating system (Model 1030, Small Animal Instruments, Inc., Stony Brook, NY, USA). Body temperature was monitored by rectal temperature probe (Small Animal Instruments, Inc), and maintained at 37 °C using an automatic heating fan.

Dynamic contrast enhanced (DCE)-MRI was obtained using a fast low angle shot imaging sequence (FLASH) with flip angle = 20°, TR = 7 ms, TE = 1.5 ms and time resolution = 4.5 s. The image matrix was 64 × 64, giving a resolution of 0.47 × 0.53 × 1.28 mm^3^. Contrast agent Gd-DOTA (Dotarem, Guerbet, Paris, France) diluted 1:10 in 0.9% NaCl, 180 μl was administered in the tail vein using an infusion pump (Harvard Apparatus, Holliston, MA, USA) roughly 60 s into imaging.

A total of 176 DCE-MRI data sets were obtained for 66 individual tumours, 48 of which had a complete set of three scans for all assessment points, 14 had two, and 4 had one. Missing data was due to failed injection (*n* = 7), tumours out of FOV (*n* = 3), software malfunction (*n* = 4), user malfunction (*n* = 4), and loss due to time constraints (*n* = 4).

### Image analysis and kinetic Modelling

Image analysis was done in IDL 8.3. Full tumours were manually delineated in axial DCE-images summed over all time frames, and relative signal increase curves (RSI = S-S_0_/S_0_, where S_0_ and S are the pre- and post-contrast signal intensities, respectively) were extracted for all voxels. RSIs were converted to Gd-DOTA concentration according to *C* [*mM*] = *RSI*/*r*_1_*T*_1_, assuming a uniform *T*_*1*_-time of 1400 ms for tumour tissue and relaxivity *r*_*1*_ at 7.05 T for Gd-DOTA of 3.70 mM^− 1^ s^− 1^ [13, 14]. Raw tumour contrast enhancement was quantified in the wash-in (30 s p.i.) and wash-out (300 s p.i.) phase in terms of the median concentration over all voxels averaged over ±3 frames (27 s).

Individual image derived arterial input functions (AIFs) were acquired by seeded growing of the left ventricle in images summed over 0–30 s p.i. The resulting volume was pared by excluding voxels with a peak-to-final frame-ratio of less than four (indicating anomalous time-intensity pattern), the median over which was fitted to a tri-exponential function using Levenberg-Marquardt least squares minimisation.

A population AIF was constructed from the median over fit parameters for all AIFs deemed as adequate (criterion for individual AIFs was at least 10 voxels with consistent peak in 1–2 time frames). As individual AIFs could not be obtained for all tumours, and since contrast agent dosing and automatic injection were standardised for all animals, the population AIF was used in the modelling of all tumours (see Additional file 1). Compared with using individual AIFs for the subset of tumours with adequate AIFs, this approach indeed reduced the variance in kinetic parameters within treatment groups (data not shown).

The time frame of injection was automatically identified for each mouse using a gradient descent method. For each mouse, the AIF (population-based) was shifted according to this time frame, and voxel-wise enhancement curves were then fitted with the extended Tofts model [4, 15] to yield parameter maps of K^trans^, ν_e_ and ν_B_. Pearson’s *r*^2^ was used to assess model fit.

### Statistical analysis

For assessment of longitudinal treatment effects, tumour type (HBCx34 vs HBCx39), treatment (Dox, GW3965 and Dox + GW3965 vs Control) and time post treatment (p. t.) (Day 1 and 6 vs Baseline) were constructed as indicator variables. Univariate repeated measures mixed-effects (RMME) maximum likelihood regression models were fitted to DCE-MRI-derived data (enhancement data, Tofts parameters and tumour volumes) in Stata/MP 15.1 (StataCorp LLC, College Station, TX, USA) with predictors treatment, time and type, and treatment×time, type×time and type×time×treatment interactions. More precisely, for each individual tumour *i* we have observations at time *t* = 0, 1, 6 following the equation

$$ {Y}_{i,t}=\sum \limits_{j=1}^d{\beta}_j{X}_{j,i,t}+{u}_i+{\varepsilon}_{i,t} $$

where covariates *X*_*j*, *i*, *t*_ are the non-random indicator variables and β_j_ the estimated coefficients. It is assumed that *u*_*i*_ and *ε*_*i*, *t*_ are zero mean normal, and that all error terms are independent. The Akaike criterion (AIC) was used to assess model suitability for all possible models with group-wise elimination, under two restrictions; (i) identical distribution for all baseline data for each tumour type (i.e. variable treatment and interaction term type×treatment at time zero were excluded) and (ii) exclusion of all higher order terms upon elimination of a lower order term.

For assessment of differences in tumour volumes, Mann-Whitney U tests were used for differences between tumour types. For testing of treatment effects, Kruskal-Wallis H tests were used. In general, *p*-values less than 0.05 were regarded as statistically significant.

## Results

**Fig. 1:**
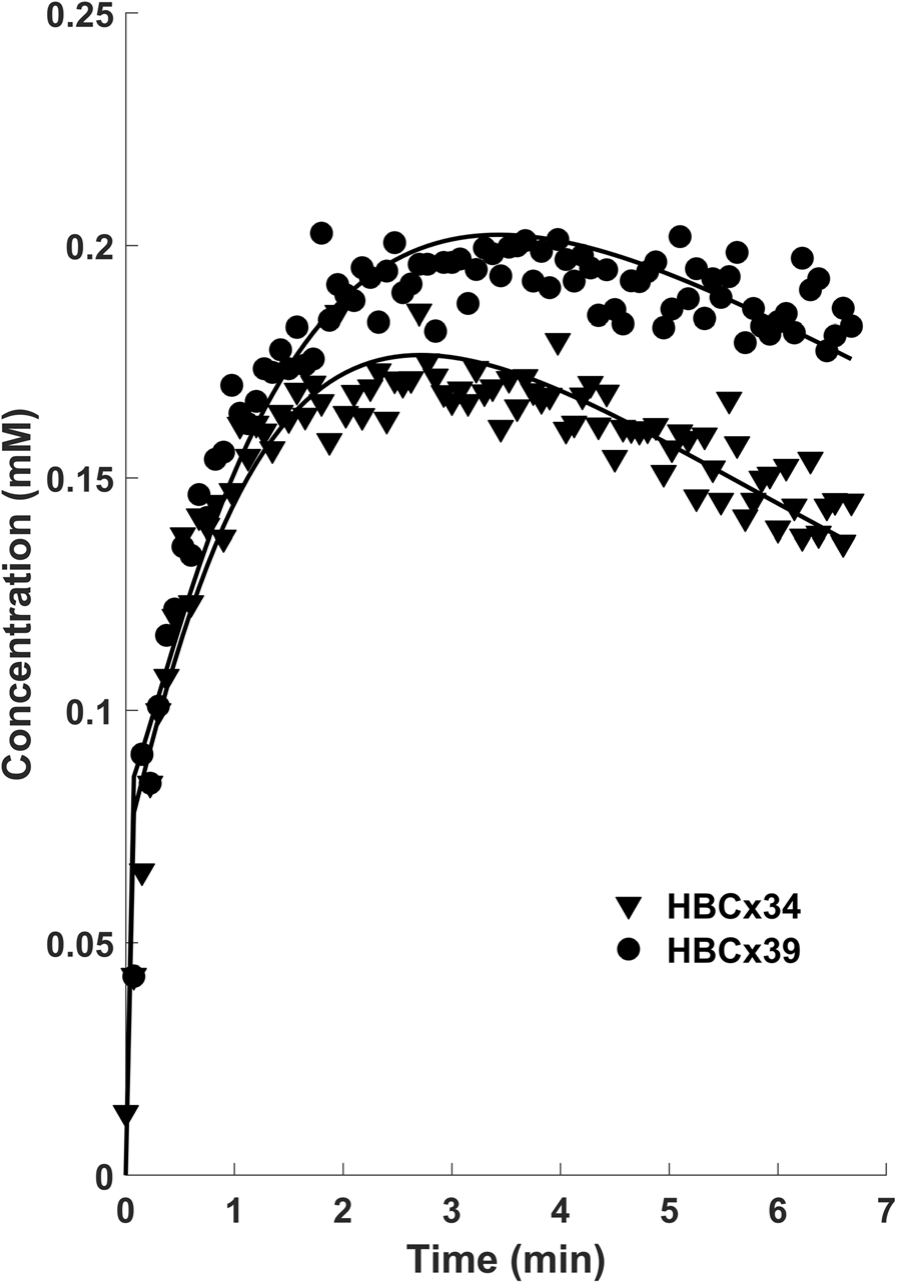
Median Gd-DOTA concentration curves fitted with the Tofts model over all HBCx39 (circles) and HBCx34 (triangles) tumours at baseline (day 0)

Mean raw concentration curves for each tumour xenograft at baseline are shown in Fig. 1. The subtypes appear to have roughly equal influx rate, however HBCx39 reached significantly greater enhancement levels after 3–4 min (*p* < 0.05) and overall curve shape differed, with HBCx34 showing more pronounced wash-out.

**Fig. 2:**
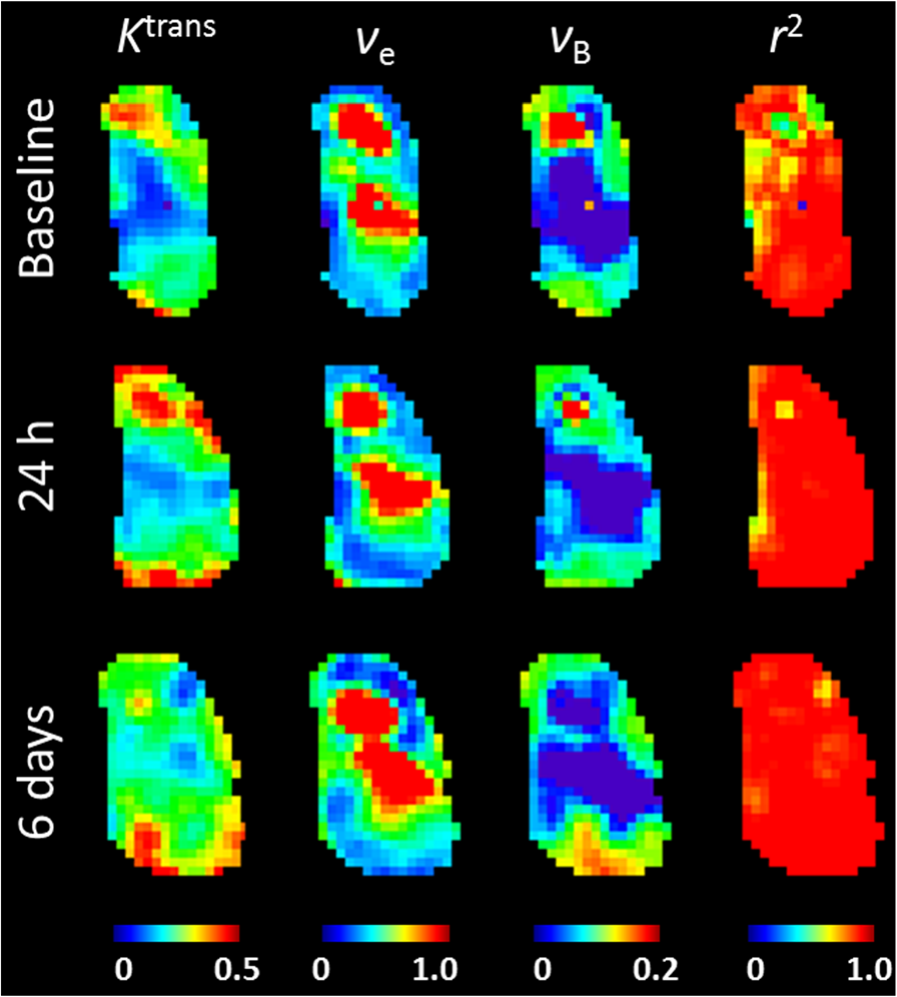
Tofts parametric images for a representative tumour treated with doxorubicin at baseline, 24 h and 6 days post treatment

The extended Tofts model with population AIF was fitted voxel-by-voxel to contrast enhancement curves for all tumours to yield parameters *K*^trans^, ν_e_ and ν_B_. Parametric images for Tofts data as well as goodness of fit (*r*^2^) for a representative doxorubicin treated HBCx34 tumour at baseline, day 1 and day 6 are shown in Fig. 2. The parametric charts are seen to be heterogeneous with clearly recognisable features over time. The central poorly perfused area at baseline shows an increase in *K*^trans^ by day 6. ν_e_ also appears to be slightly increased on day 6. Kinetic model fit was overall good with median (range) of 0.82 (0.27–0.96).

**Fig. 3:**
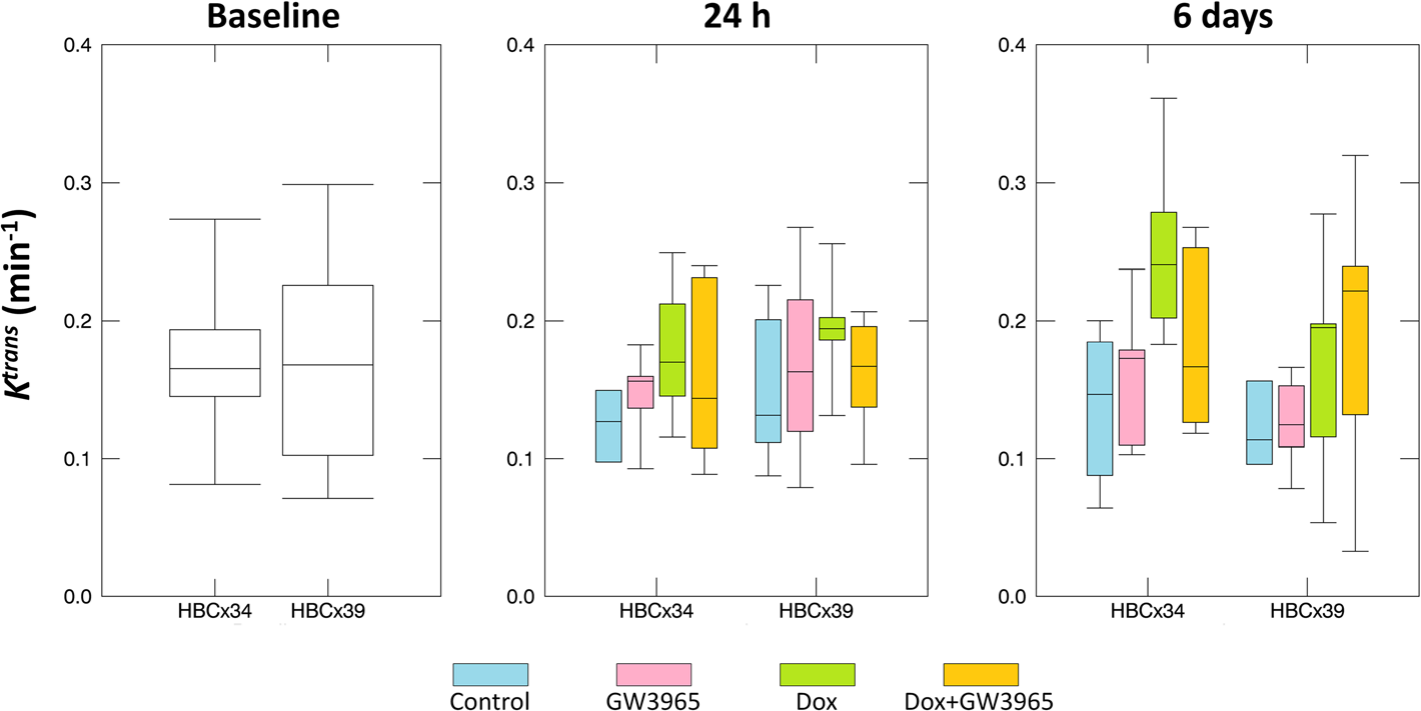
*K*^trans^ raw data for each treatment group for HBCx34 and HBCx39 tumours at baseline, 24 h and 6 days post treatment

For each tumour, the median over all voxels for each metric was taken as representative. Early enhancement was highly correlated with vascular Tofts parameters *K*^trans^ (*r* = 0.88) and ν_B_ (*r* = 0.92), both with *p* < 0.0001. At baseline, perfusion-related wash-in enhancement, *K*^trans^ and ν_B_ did not differ significantly between xenograft subtypes (*p* > 0.3), however clearance-related wash-out enhancement and ν_e_ were significantly greater for HBCx39 (*p* = 0.005 and *p* = 0.0008). Upon inspection of data for each treatment group as a function of time (see Fig. 3 for *K*^trans^, Additional file 1: Figures S3 and S4 for other data), we observed no decisive trends with either treatment or time. In order to analyse treatment effects on longitudinal DCE-MRI derived metrics, taking into account individual tumour (within-subject) variability, all possible univariate RMME models were applied to median tumour data. The AIC was used for model selection; wash-in enhancement, *K*^trans^ and ν_B_ all preferred models including treatment effect with time (see Additional file 1*).* ν_e_, wash-out enhancement and image-derived tumour volume preferred simpler models taking only tumour type and time without interactions into account. Estimated coefficients, standard errors and associated *p*-values are shown in Additional file 1: Table S1.

**Fig. 4:**
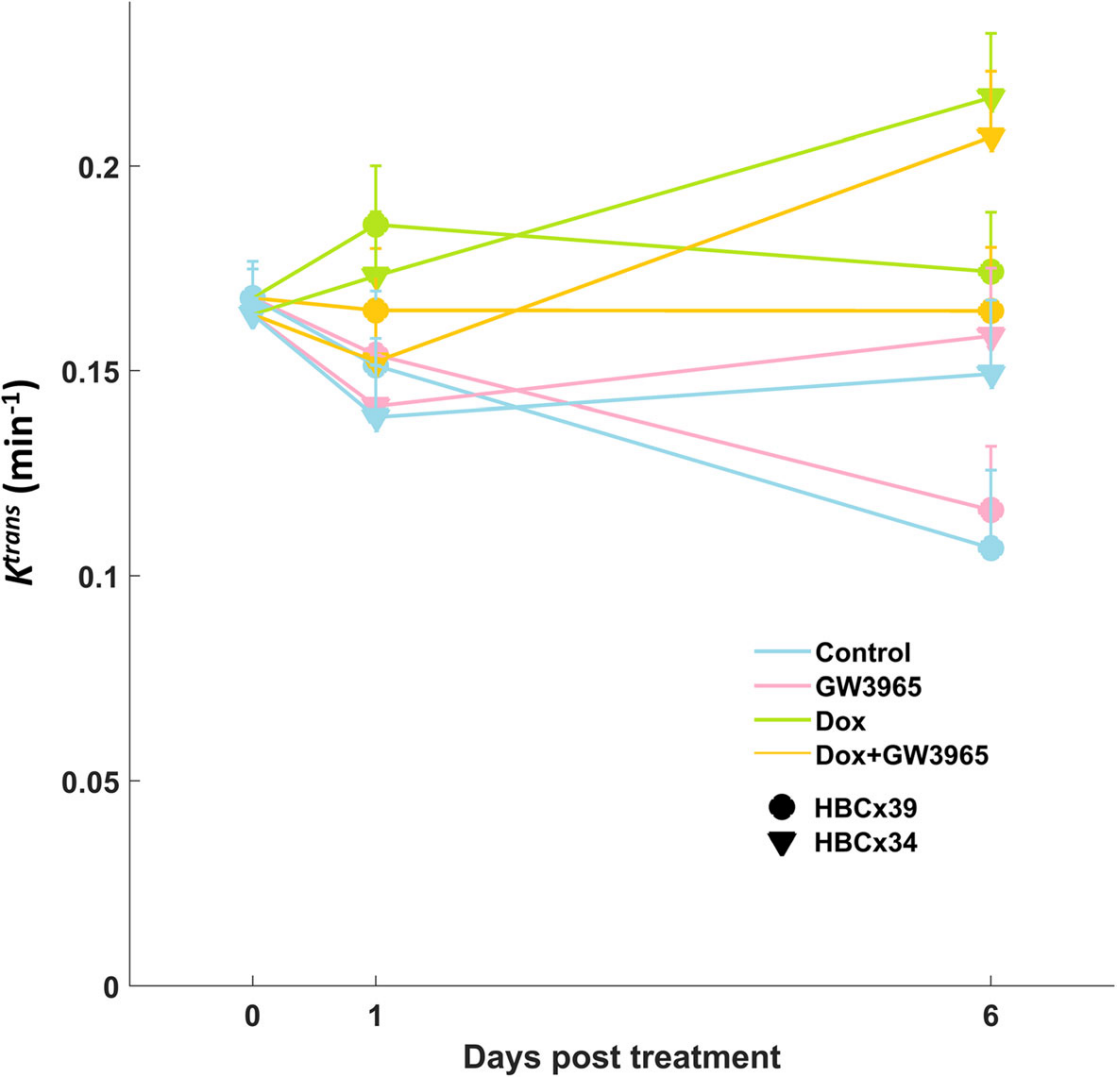
*K*^trans^ model estimates for each tumour type and treatment at baseline, 24 h and 6 days post treatment. Standard error bars are given in the positive direction

RMME models found a significant impact of doxorubicin treatment on all perfusion-related data, estimating a significant increase on day 6. However, there was also a concurrent significant decline in perfusion-related metrics with time for HBCx39 tumours, counteracting the treatment related effect, resulting in no estimated change in *K*^trans^, ν_B_ and wash-in enhancement (*p* > 0.4) on day 6 relative to baseline for these tumours. For HBCx34 however, there was a significant increase of ~ 30% for early enhancement (*p* < 0.011) and *K*^trans^, (*p* < 0.017) and 40–50% for ν_B_ (*p* < 0.024) estimated by day 6. See Fig. 4 for estimated treatment effects on *K*^trans^. In general, treatment with doxorubicin alone and in combination with GW3965 had approximately the same significant positive effect on all vascular metrics on day 6 (*p* > 0.7 that they differ). On the other hand, treatment with GW3965 had no significant impact on any metric, and GW3965 treated tumours did not differ significantly from controls on day 6 (*p* > 0.7 for all metrics).

Wash-out enhancement and ν_e_ were overall significantly less for HBCx34 tumours as compared with HBCx39. There were no significant differences with treatment, but six days after therapy, ν_e_ was significantly increased (*p* = 0.026). Image derived tumour volumes increased with time and were significantly greater on day 6 compared with baseline (*p* < 0.001).

## Discussion

In this study we investigated whether DCE-MRI and pharmacokinetic modelling may be used to detect and differentiate ultra-early effects of chemo- and targeted therapies in two different breast cancer models. Significant effects on tumour contrast wash-in and pharmacokinetic parameters from doxorubicin treatment was found after 6 days, but not 1 day after treatment. Conversely, no significant change in tumour volume was associated with treatment.

Doxorubicin has been shown to have severe effect on vessels shortly after administration (2–5 min) [8]. We thus hypothesised that there could be some impact on tumour perfusion 24 h after treatment, but found no significant effects at this time point. There were small estimated increases associated with doxorubicin (but not the combination therapy) on wash-in phase enhancement and *K*^trans^, but these did not reach statistical significance. This would indicate that 24 h is too early to evaluate effects of cytotoxic treatment by DCE-MRI. On day 6 however, treatment with doxorubicin, both as mono- and combination therapy, had a significant impact on all metrics reflecting tumour vasculature (wash-in enhancement, *K*^trans^ and ν_B_). Model coefficient estimates for Dox and Dox + GW3965 were virtually indistinguishable across all metrics, indicating that most if not all of the effects seen were attributable to doxorubicin. Consistently, estimated coefficients for GW3965 were more or less the same as those for the reference group, indicating no apparent effects of this treatment. It may be that there were no effects at all over the duration of the study, or that potential effects were not detectable by DCE-MRI. A recent study reported a significantly smaller anti-tumour effect of LXR-agonism in immunodeficient mice compared with immunocompetent counterparts [16], indicating that the latter animal model should be investigated in future LXR agonist response studies with DCE-MRI.

The two subtypes behaved differently with time; HBCx39 tumours had no estimated change in wash-in enhancement, *K*^trans^ or ν_B_, whereas HBCx34 had significant estimated increases in these metrics relative to baseline. This could be a result of increased permeability caused by vascular damage, which has been seen after radiotherapy [17] and could possibly give an apparent increase in *K*^trans^. However, increased perfusion early after anti-angiogenic treatment is also thought to reflect vascular normalisation [18]. A recent study found increased *K*^trans^ 2–8 days following anti-angiogenic treatment, which was significantly correlated with vessel maturity index in a glioma tumour model [19]. Doxorubicin has been found to repress VEGF-expression in vitro [20]. Pruning of immature, leaky vessels would result in improved flow in more established vasculature and an apparent increase in perfusion. The observed effect on DCE-MRI parameters may thus reflect vascular normalisation following this anti-angiogenic element of doxorubicin. Both *K*^trans^ and ν_B_ expressed this effect; however, the increase in ν_B_ is more likely reflective of a flow component to the parameter as opposed to increased vessel volume.

Regarding increase in *K*^trans^ following chemotherapy, we recently saw a similar response in a dynamic ^18^F-FDG-PET study of different breast cancer subtypes including the ones studied here [21]. In that work, HBCx39 were poor responders to therapy (in terms of volume regression) and displayed no change or a decline in perfusion related metrics (K_1_ and SUV_E_) on day 10 pt. relative to baseline, whereas HBCx34 overall increased in perfusion and had high response rate. This is in line with what we observed in the present study, and our results indicate that the effects on perfusion seen on day 10 pt. are already manifested by day 6 pt. However, the PET study found no significant changes 3 days pt., in line with that we currently found no effects on *K*^trans^ 24 h pt. The vascular normalisation window might not yet be open on day 1 or 3, or the effects still too small relative to the measurement variability.

In the present work, we observed no significant changes in tumour volume with treatment. Calliper measurements of tumours agreed well with DCE-MRI derived volumes (*r* = 0.88, *p* < 0.0001, data not shown). Volume increased with time and was overall significantly larger on day 6. When fitted with the larger (non-preferred) model allowing for treatment effects, there were small decreases for tumours receiving doxorubicin alone or in combination, but these were not statistically significant (*p* = 0.82 for Dox and *p* = 0.22 for Dox + GW3965). Thus, it is too early to see significant volumetric effects of doxorubicin 6 days pt. in these tumours.

Estimated percentage change relative to baseline after Dox (mono and combo) treatment in HBCx34 tumours was on the same scale for wash-in enhancement and *K*^trans^. ν_B_ had the greatest estimated percentage increase, but variability was also greatest in this parameter with test-retest mean relative differences (±SD) of 60 ± 27% compared with 16 ± 12% for *K*^trans^ for the control group in scans taken 24 h apart.

From a monitoring perspective, where one is simply interested in a bulk biomarker to advise clinical decisions, early enhancement (obtainable by a quick acquisition before and after CA administration) was equally informative as the more complex modelling. However, pharmacokinetic modelling allowed us to identify the effect as one of increased perfusion as opposed to increased EES and retention. Considering estimated physiological changes unrelated to treatment, there was a significant decline in *K*^trans^ attributed to the passage of time for HBCx39 tumours. This could be due to increasing interstitial fluid pressure (IFP), associated with elevated microvascular hypertension resulting from angiogenesis and increasingly abnormal tumour vasculature [22, 23]. IFP has been found to be inversely correlated with *K*^trans^ in DCE-MRI with other small molecular weight contrast agents [24]. As discussed above, HBCx39 has been shown to respond poorly to chemotherapy in comparison with HBCx34 [25], in line with that declining *K*^trans^ may reflect a more treatment resistant phenotype. There was also a decline in ν_B_ with time, trending for HBCx34, but significant for HBCx39, which could suggest that angiogenesis is not keeping up with tumour growth. However, there was also a significant increase in ν_e_ by day 6 pt., suggestive of an increase in necrotic fraction [26]. Some of the effect could thus be due to increasing necrosis supplanting vascular volume. The EES was overall significantly larger for HBCx39, indicating greater levels of necrosis in these tumours.

## Conclusions

In conclusion, we could not detect treatment effects by DCE-MRI in our experimental breast cancer models 1 day post treatment. After six days however, doxorubicin treatment had significant impact on perfusion as measured by *K*^trans^ and ν_B_.

## Supplementary information

**Additional file 1: Figure S1.** All adequate AIFs for 90 animals. The median AIF used in the Tofts modelling of all tumours is drawn in blue. **Figure S2.** Sample DCE-MRI data for four different tumours. Pre-contrast T1 weighted central axial image with the tumour delineated (left), parametric maps of K^trans^, ν_e_ and ν_B_ and r^2^ (middle), and time-concentration curves with Tofts fitted model for median data representing the tumour (right). **Figure S3.** DCE-MRI derived data observed for HBCx34 (left column(s)) and HBCx39 (right column(s)) tumours at baseline, 1 and 6 days posttreatment; Gd-DOTA concentration in the A wash-in and B wash-out phase C νe, D νB and E tumour volume based on DCE-MRI images. **Figure S4.** RMME model estimates with standard error bars shown in the positive direction. **Table S1.** Estimated coefficients with standard errors and *p*-values for univariate RMME of DCE-MRI derived data. Grey outlines indicate significant (*p* < 0.05) covariates.

## Data Availability

The datasets generated during and/or analysed during the current study are available from the corresponding author on reasonable request.
