# Supplementary material for: Ultra-early changes in vascular parameters from dynamic contrast enhanced MRI of breast cancer xenografts following systemic therapy with doxorubicin and liver X receptor agonist
Source: Cancer Imaging. 2019 Dec 19;19:88. doi: 10.1186/s40644-019-0280-y (PMC6924064; doi:10.1186/s40644-019-0280-y)
Supplement: Supplementary file 1 — Additional file 1: Figure S1. All adequate AIFs for 90 animals. The median AIF used in the Tofts modelling of all tumours is drawn in blue. Figure S2. Sample DCE-MRI data for four different tumours. Pre-contrast T1 weighted central axial image with the tumour delineated (left), parametric maps of Ktrans, νe and νB and r2 (middle), and time-concentration curves with Tofts fitted model for median data representing the tumour (right). Figure S3. DCE-MRI derived data observed for HBCx34 (left column(s)) and HBCx39 (right column(s)) tumours at baseline, 1 and 6 days posttreatment; Gd-DOTA concentration in the A wash-in and B wash-out phase C νe, D νB and E tumour volume based on DCE-MRI images. Figure S4. RMME model estimates with standard error bars shown in the positive direction. Table S1. Estimated coefficients with standard errors and p-values for univariate RMME of DCE-MRI derived data. Grey outlines indicate significant (p < 0.05) covariates. [file 40644_2019_280_MOESM1_ESM.docx]

Additional file 1

**Ultra-early changes in vascular parameters from dynamic contrast enhanced MRI of breast cancer xenografts following systemic therapy with doxorubicin and liver X receptor agonist**

Kathinka E. Pitman^1,2^, Kine M. Bakke^1,3^, Alexandr Kristian^4^, Eirik Malinen^1,2^

*^1^Department of Physics, University of Oslo, Oslo, Norway; ^2^Department of Medical Physics/ ^4^Department of Tumour Biology Oslo University Hospital, Oslo, Norway; ^3^Department of Oncology, Akershus University Hospital, Lørenskog, Norway*

## Extended ‘Tofts’ Modelling

As described in the main manuscript, individual image derived arterial input functions (AIFs) were acquired by seeded growing of the left ventricle during the first pass of tracer. Voxels indicating anomalous time-intensity pattern were excluded, and the median over the remaining voxels, take at each time point, was fitted to a tri-exponential function using Levenberg-Marquardt least squares minimisation. For some animals, the AIF could not be extracted either due to failed seeded growing or that few of the grown voxels showed adequate time-enhancement patterns, A population AIF was constructed from the median over fit parameters for all animals where the AIF could be estimated (Figure 1).


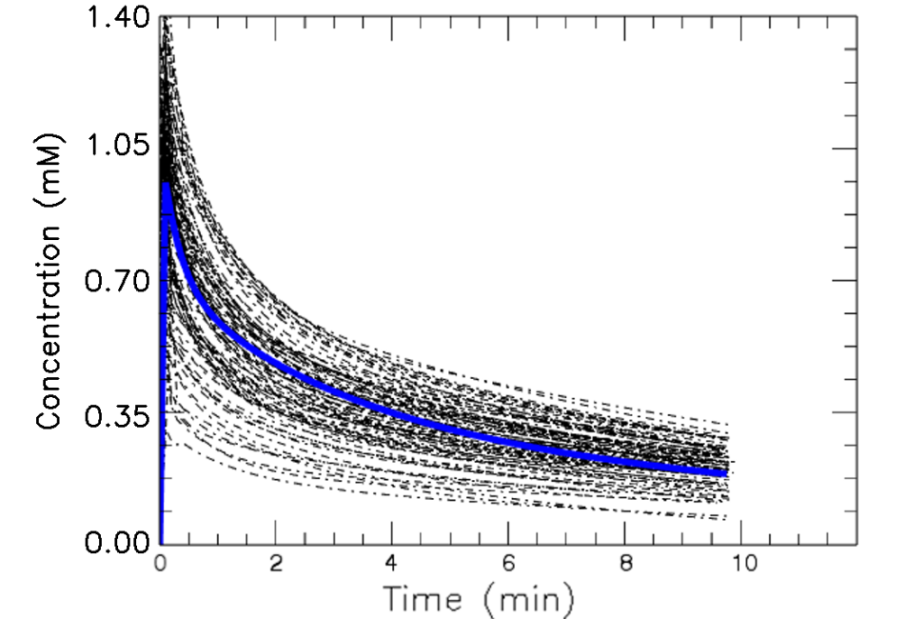


Figure S1 - All adequate AIFs for 90 animals. The median AIF used in the Tofts modelling of all tumours is drawn in blue.

Shown in Figure 2 are extended Tofts modelling data for a set of example tumours. Obtained parametric charts for *K*^trans^, ν_e_ and ν_B_ as well as model fit assessed by *r*^2^ are shown together with a median dynamic uptake curve for each tumour.


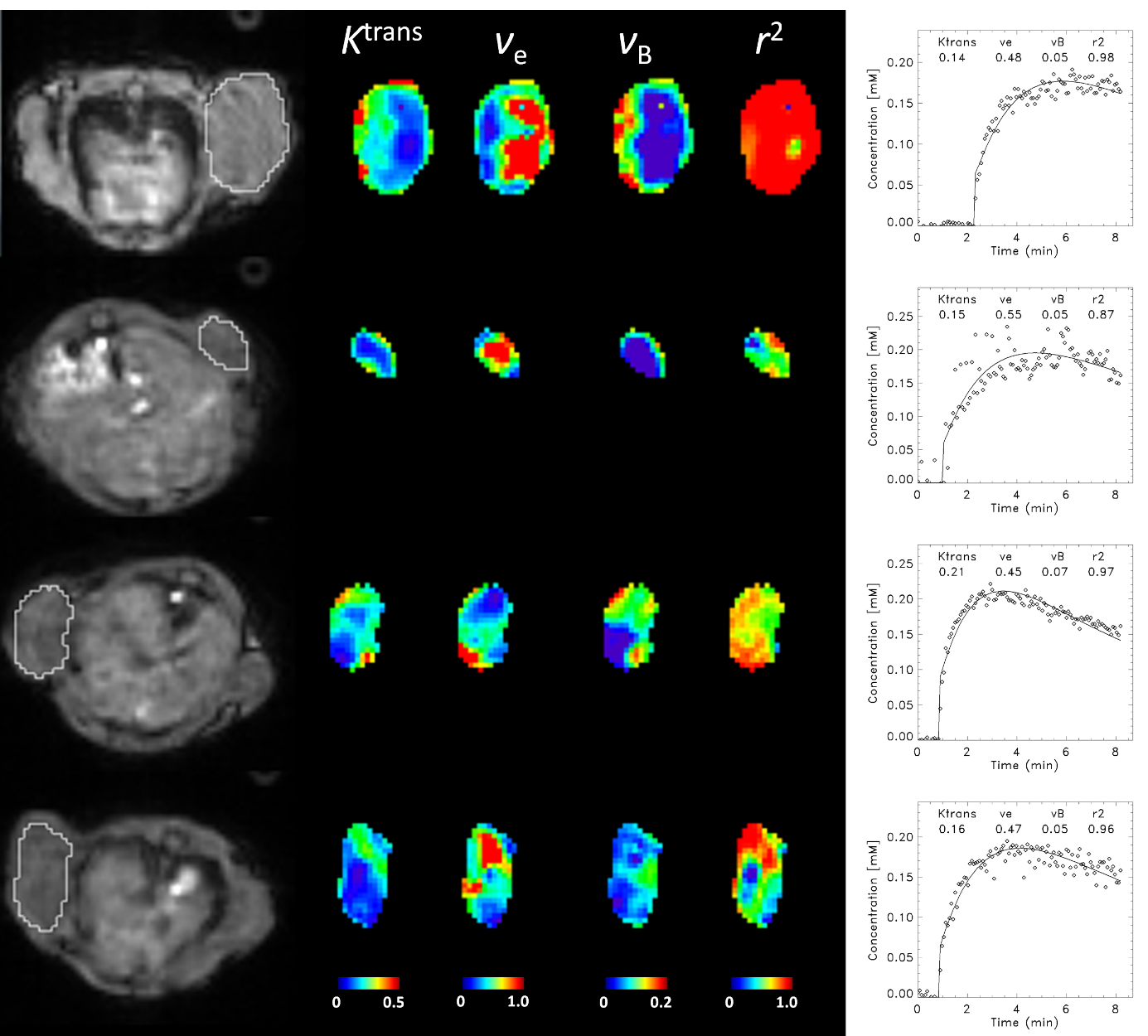


Figure S2 - Sample DCE-MRI data for four different tumours. Pre-contrast T1 weighted central axial image with the tumour delineated (left), parametric maps of K^trans^, ν_e_ and ν_B_ and r^2^ (middle), and time-concentration curves with Tofts fitted model for median data representing the tumour (left).

## Additional monitoring data

Tumours were randomly assigned to treatment with doxorubicin (Dox), LXR-agonist (GW3965), combination therapy (Dox+GW3965) or no treatment (Control). Figure 3 shows raw data acquired from dynamic enhancement curves (wash-in and wash-out phase contrast agent concentrations) and Tofts modelling (ν_e_ and ν_B_), as well tumour volumes. *K*^trans^ data is shown in the main manuscript.

**
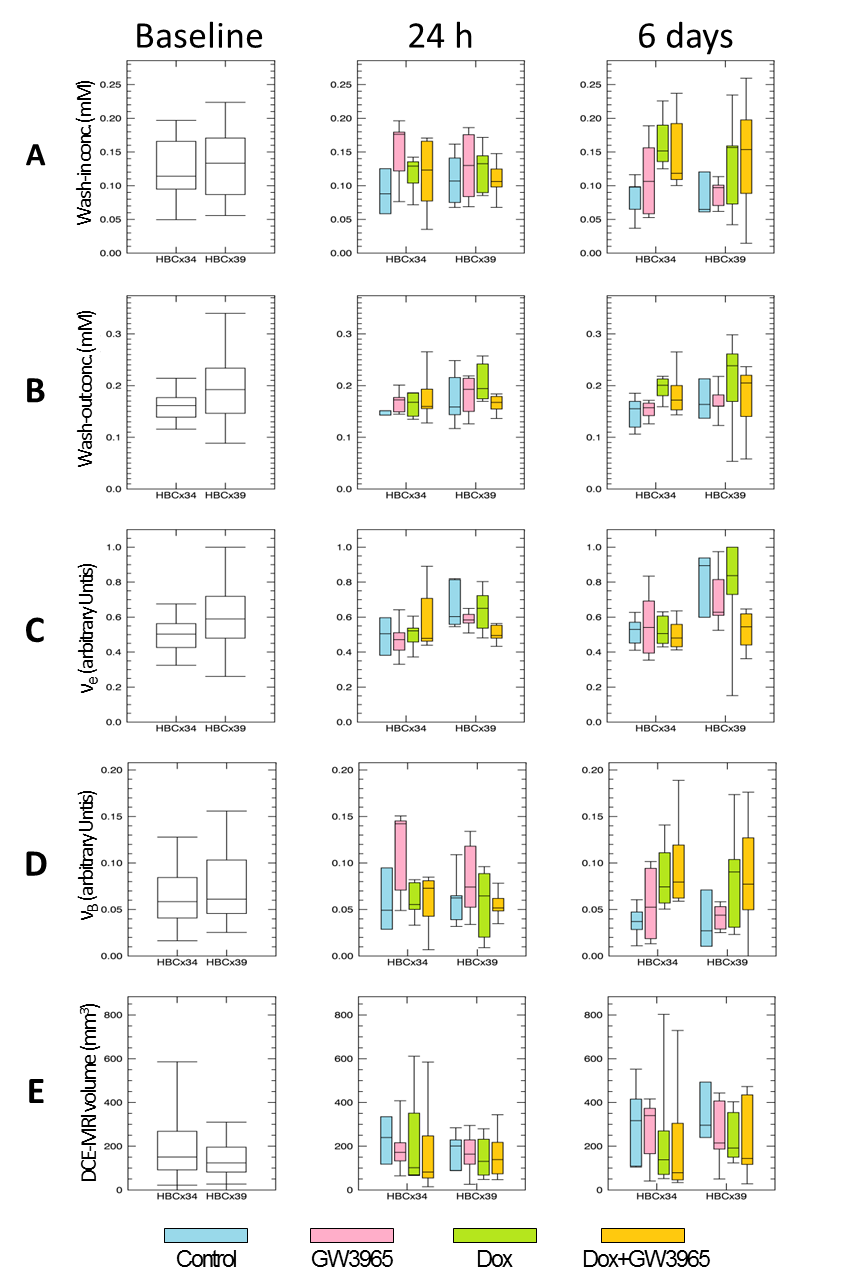
**

Figure S3 - DCE-MRI derived data observed for HBCx34 (left column(s)) and HBCx39 (right column(s)) tumours at baseline, 1 and 6 days posttreatment; Gd-DOTA concentration in the (A) wash-in and (B) wash-out phase (C) νe, (D) νB and (E) tumour volume based on DCE-MRI images.

**Repeated measures mixed-effects maximum likelihood regression**

For analysis of treatment effects in the longitudinal data panel, we applied univariate repeated measures mixed-effects (RMME) models to all metrics, using the Akaike criterion (AIC) for model selection in each case. Estimated coefficients for covariates and interaction terms prescribed by the AIC are shown in Table 1 for each metric.

*Table 1* - Estimated coefficients with standard errors and p-values for univariate RMME of DCE-MRI derived data. Grey outlines indicate significant (p<0.05) covariates.

Wash-in phase concentration and ν_B_ preferred models accounting for treatment effects with time which differed for subtypes, whereas no treatment effects were estimated for ν_e_. Wash-out phase concentration was significantly different between subtypes but was not estimated to change with time, whereas tumour volumes were only estimated to be impacted by time. Estimated developments with time are shown for each metric in Figure 4.


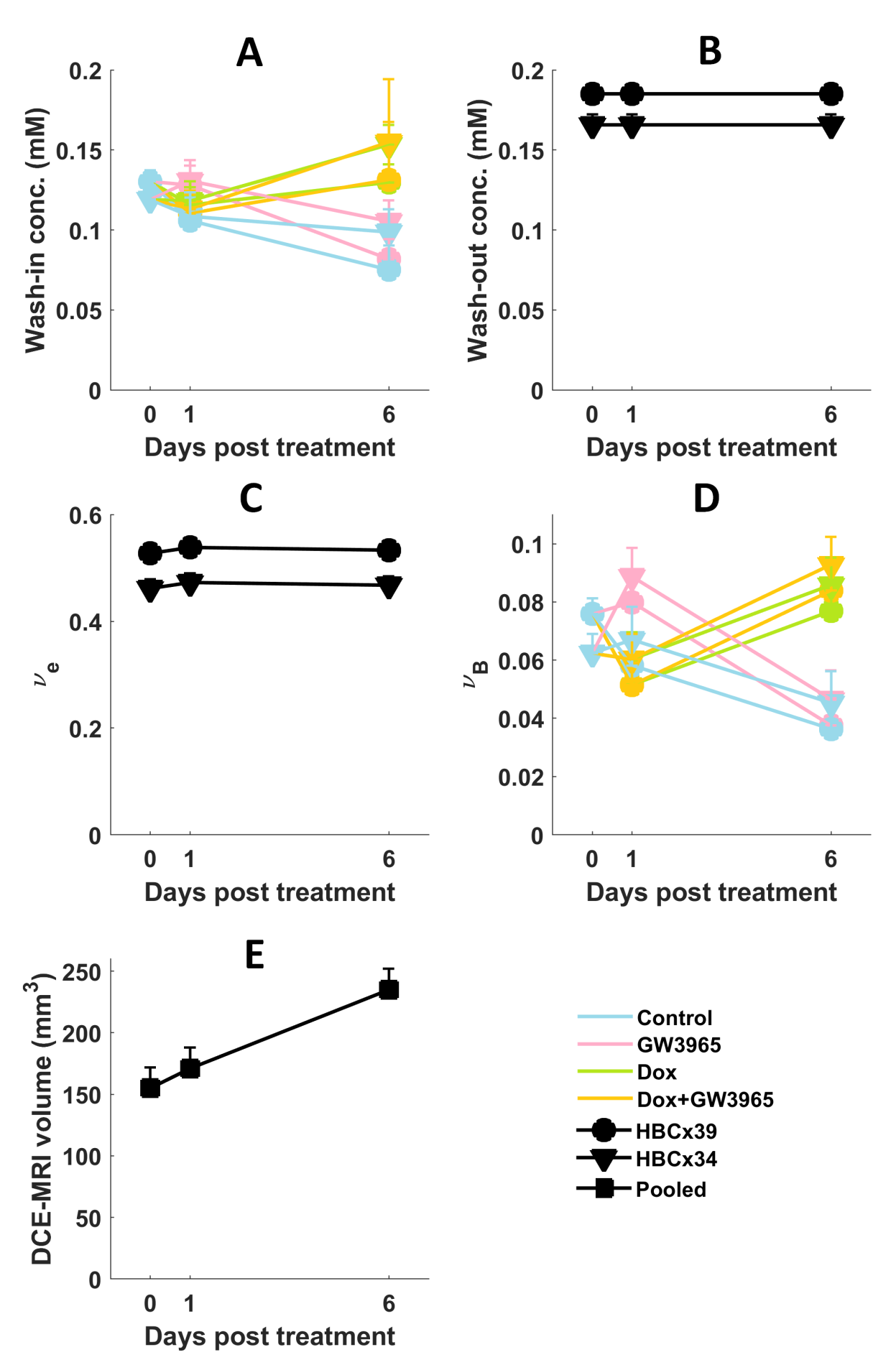


Figure S4 – RMME model estimates with standard error bars shown in the positive direction.
